# Identification of key genes associated with polycystic ovary syndrome (PCOS) and ovarian cancer using an integrated bioinformatics analysis

**DOI:** 10.1186/s13048-022-00962-w

**Published:** 2022-02-28

**Authors:** Juan Zou, Yukun Li, Nianchun Liao, Jue Liu, Qunfeng Zhang, Min Luo, Jiao Xiao, Yanhua Chen, Mengjie Wang, Kexin Chen, Juan Zeng, Zhongcheng Mo

**Affiliations:** 1 Department of Obstetrics and Gynecology, The Second Affiliated Hospital of University of South China, University of South China, Hengyang, Hunan China; 2 Hunan Province Key Laboratory of Tumor Cellular & Molecular Pathology, Cancer Research Institute, University of South China, Hengyang, Hunan China; 3 Clinical Anatomy & Reproductive Medicine Application Institute, Department of Histology and Embryology, University of South China, Hengyang, Hunan 421001 People’s Republic of China; 4 Department of Endocrinology, The Affiliated Nanhua Hospital, University of South China, Hengyang, Hunan China; 5 Institute of Basic Medical Sciences, College of Basic Medicine, Guilin Medical University, Guilin, Guangxi China; 6 Department of Laboratory Medicine, The Affiliated Nanhua Hospital, University of South China, Hengyang, Hunan China; 7 Department of Anesthesiology, The Second Affiliated Hospital of University of South China, University of South China, Hengyang, Hunan China

**Keywords:** Ovarian cancer, Polycystic ovary syndrome, Bioinformatic analysis, OGN, Prognostic marker

## Abstract

**Background:**

Accumulating evidence suggests a strong association between polycystic ovary syndrome (PCOS) and ovarian cancer (OC), but the potential molecular mechanism remains unclear. In this study, we identified previously unrecognized genes that are significantly correlated with PCOS and OC via bioinformatics.

**Materials and methods:**

Multiple bioinformatic analyses, such as differential expression analysis, univariate Cox analysis, functional and pathway enrichment analysis, protein–protein interaction (PPI) network construction, survival analysis, and immune infiltration analysis, were utilized. We further evaluated the effect of OGN on FSHR expression via immunofluorescence.

**Results:**

TCGA-OC, GSE140082 (for OC) and GSE34526 (for PCOS) datasets were downloaded. Twelve genes, including RNF144B, LPAR3, CRISPLD2, JCHAIN, OR7E14P, IL27RA, PTPRD, STAT1, NR4A1, OGN, GALNT6 and CXCL11, were identified as signature genes. Drug sensitivity analysis showed that OGN might represent a hub gene in the progression of PCOS and OC. Experimental analysis found that OGN could increase FSHR expression, indicating that OGN could regulate the hormonal response in PCOS and OC. Furthermore, correlation analysis indicated that OGN function might be closely related to m6A and ferroptosis.

**Conclusions:**

Our study identified a 12-gene signature that might be involved in the prognostic significance of OC. Furthermore, the hub gene OGN represent a significant gene involved in OC and PCOS progression by regulating the hormonal response.

**Supplementary Information:**

The online version contains supplementary material available at 10.1186/s13048-022-00962-w.

## Introduction

Polycystic ovary syndrome (PCOS), a multisystem reproductive metabolic disease of the reproductive system, is characterized by the pathological accumulation of nonmaturating and atretic follicles, ovarian and stromal abnormal hyperplasia, hyperandrogenaemia (HA), hyperinsulinaemia, insulin resistance (IR), aberrant metabolism, an imbalance in the ratio of luteinizing hormone (LH) to follicle-stimulating hormone (FSH), and polycystic ovaries [1]. The mRNA and microRNA profiles of PCOS patients were extremely similar to ovarian cancer (OC) patients, indicating that the same molecular mechanisms might be involved in OC and PCOS patients [2, 3].

During PCOS progression, HA is an important factor for promoting ovulatory dysfunction [4], increasing the frequency and amplitude of LH and GnRH pulse secretion [5], inducing lipid metabolism disorders [6], mediating hyperinsulinaemia and insulin resistance [7], and dysregulating the ratio of LH to FSH [8].

For the clinical management of patients with PCOS, anti-androgen therapy is the first line of treatment for patients diagnosed with PCOS [9]. Over recent years, the relationship between PCOS and OC progression has been a hot topic for these studies because the AR signalling axis and metabolic disorders are correlated with a high risk of OC [3, 10, 11]. Both OC and PCOS are multifactorial diseases with genetic, endogenous, endocrine-maladjusted, metabolically disturbed and environmental factors. Therefore, a better understanding of the physiopathologic mechanism regulating these complex molecular effects is urgently needed to promote the research and development of new drugs and to improve these patients’ prognoses.

With the development of bioinformatic analysis and public databases, such as The Cancer Genome Atlas (TCGA) [12] and Gene Expression Omnibus (GEO) [13], understanding the molecular mechanisms of currently available treatments against PCOS and OC provides a means to emphasize targets for effective treatments. For example, Surleen Kaur found that in PCOS tissues, certain differentially expressed genes correlated with metabolic disorders and oxidative stress and exhibited a potential relationship with cancer [14]. HSA2 and CBLN1 were all identified in a PCOS dataset [15]. Another study identified 36 highly altered genes, among which 10 were common to endometrial cancer (EC), OC and breast cancer (BC), promoting cell proliferation, hormone response, and endogenous stimulation [16]. A series of bioinformatics tools were used for integrated analysis and detection of metabolism-related genes (MRGs) in OC. For example, we found that ENPP1, FH, CYP2E1, HPGDS, ADCY9, NDUFA5, ADH1B and PYGB were correlated with the underlying mechanisms of metabolic reprogramming in OC progression [17]. Yang et al. found that CCNB2, TYMS, KIF11, KIF4A, BIRC5, BUB1B, FOXM1, and CDC20 might represent potential therapeutic targets for OC patients [18]. Nevertheless, whether these hub genes are uniquely involved in individual disease progression remains unclear.

To determine the potential molecular mechanisms between PCOS and OC, we integrated two datasets, namely, PCOS and OC. Utilizing multiple bioinformatic and experimental analyses, we sought to validate hub genes and pathways of interest and to search for potential therapeutic drugs or targets in PCOS and OC.

## Materials and methods

### Data extraction

**The** TCGA database [12] (https://portal.gdc.cancer.gov/) is the largest cancer gene information database and includes data concerning gene expression. We extracted data for 374 cases of OSC patients. Moreover, we downloaded level three FPKM data for subsequent analysis. The transcriptome RNA-sequencing and clinical information of 88 normal ovarian samples were extracted from the GTEX database (https://www.gtexportal.org/) [19]. Furthermore, the GSE140082 and GSE34526 datasets were downloaded from the GEO database [13].

### Functional enrichment analyses

Functional enrichment analyses were also performed as previously published [17]. The DAVID database (https://david.ncifcrf.gov/) [20] is mainly used to perform functional and pathway enrichment analyses of differentially expressed genes and is a very good tool used by many researchers. We submitted the 128 common DEGs to the DAVID database and performed GO function and KEGG pathway analyses.

### PPI network construction

GeneMANIA (http://genemania.org) [21] is an extremely interactive online analysis site based on proteomics and genomics data that is used to construct the PPI network for 128 common DEGs associated with PCOS and OC patients.

### Establishing prognostic indicators based on DEGs

Univariate Cox analysis was used to select genes associated with prognosis, and a prognostic correlation model was further constructed. After incorporating the expression value of each specific gene, a risk score formula was constructed for each patient. According to the risk score formula, patients were divided into a low-risk group and a high-risk group using the median risk score as the cut-off point. Kaplan–Meier analysis was used to evaluate the survival difference between the two groups, and the log-rank statistical method was used for comparison. Finally, receiver operating characteristic (ROC) curves were used to study the accuracy of model prediction.

### OGN protein expression based on bioinformatic analysis

OGN protein expression in OC was confirmed using the HPA database (https://www.proteinatlas.org/) [22] and CPTAC database (https://cptac-data-portal.georgetown.edu/) [23].

### The relationship between key genes and immune infiltration

The correlation between immune cell content and the expression level of 5 key genes (JCHAIN, CXCL11, OGN, STAT1, and GALNT6) was confirmed using the TIMER database (https://cistrome.shinyapps.io/timer/) [24].

### GSVA and GSEA

Gene set variation analysis (GSVA) is a nonparametric and unsupervised method for assessing the enrichment of transcriptome gene sets. GSVA converts gene-level changes into pathway-level changes by comprehensively scoring the sets of genes of interest to judge the biological function of the samples. In this study, gene sets will be downloaded from The Molecular Signatures Database (v7.0), and each gene set will be comprehensively scored using the GSVA algorithm to evaluate the potential biological function changes of different samples.

GSEA uses a predefined set of genes, orders genes according the level of differential expression in the two types of samples, and then tests whether the predefined set of genes is enriched at the top or bottom of the sequencing table. In this study, the possible molecular mechanism of the difference in prognosis of different ovarian cancer patients was explored by comparing the differences in signal pathways between the high-expression group and the low-expression group using GSEA. Specifically the number of replacements was set to 1000, and the replacement type was set to phenotype.

### Cell culture and transfection

Human ovarian cancer cell lines (SKOV3 and KGN) were purchased from the American Type Culture Collection (ATCC, VA, USA). Dulbecco’s modified Eagle’s medium (DMEM) containing 10% (v/v) foetal bovine serum (FBS; Gibco, Invitrogen, Carlsbad, CA, USA) and 1% penicillin/streptomycin (GIBCO, CA, USA) growth media was used to culture SKOV3 and KGN cells. All cells were incubated at 37 °C and 5% CO2. OGN overexpression and empty vector plasmids were purchased from GeneCopoeia Biotechnology (GeneCopoeia Biotechnology, MD, USA). For transient cell transfection, SKOV3 and KGN cells were seeded in 6-well plates for 24 h. After incubation, cells were transfected with 3 μg empty vector and 3 μg OGN overexpression plasmid using Lipofectamine 3000 (Invitrogen, Carlsbad, CA, USA) according to the protocol to establish a cell line with upregulated OGN expression.

### qRT–PCR analysis

Total RNA was extracted by TRIzol (Invitrogen, CA, USA) according to the manufacturer’s instructions. cDNA was produced using a reverse transcription kit (TaKaRa, Dalian, China). PCR was performed using an ABI 7500 fast system (Applied Biosystems, CA, USA). Primer sequences were as follows: OGN Forward, 5´-TCTACACTTCTCCTGTTACTGCT-3´; OGN Reverse, 5´-GAGGTAATGGTGTTAT TGCCTCA-3´.

### Immunofluorescence

The immunofluorescence assays were performed with anti-FSHR (Abcam, 1:300) according to the manufacturer’s protocol. The primary antibody used in this study was against FSHR (ab113421). The cells were incubated with the corresponding FITC-conjugated secondary antibodies (Abcam, 1:200). Two hours later, 0.1% DAPI was used to stain the nucleus for 30 min. Images were detected by confocal microscopy (Leica, Jena, Germany).

### Statistical analysis

All statistical analyses were performed in the R language (Version 3.6). All statistical tests were bilateral, and P < 0.05 was considered statistically significant.

## Results

### Identification of 128 common significant differentially expressed genes (DEGs) in PCOS and OC

**Fig. 1:**
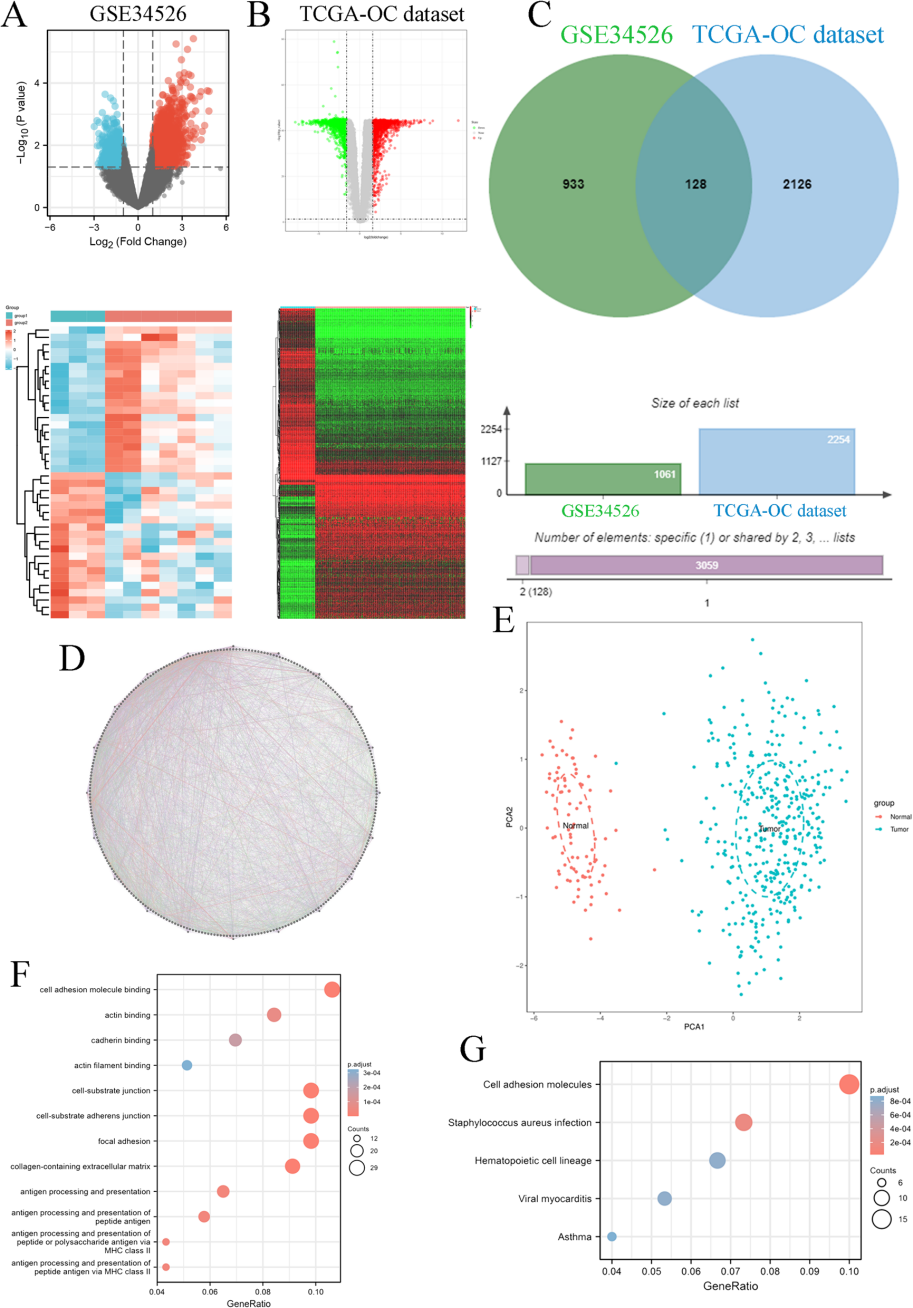
Identification of DEGs associated with PCOS and OC. **A** The DEGs in PCOS based on GSE34526 datasets. **B** The DEGs in OC based on TCGA-OC datasets. **C** The common DEGs in PCOS and OC. **D** The PPI network of 128 common DEGs in PCOS and OC. **E** The PCA between OC patient samples (TCGA-OC dataset) and normal ovary samples (GTEx-ovary datasets) based on 128 DEGs. **F** GO functional enrichment analysis for 128 DEGs. **G** KEGG enrichment analysis for 128 DEGs

First, we found 1061 DEGs in the PCOS patients compared to normal women based on the GSE34526 dataset of the GEO database and 2254 DEGs in the OC patient samples compared to normal ovary samples based on the OC dataset of the TCGA database (Fig. 1A&B). Moreover, we found 128 common DEGs in PCOS and OC progression (Fig. 1C). We also constructed a protein–protein interaction (PPI) network to identify all 120 genes in the PCOS and OC datasets. These networks were visualized using the GeneMANIA database (Fig. 1D), which revealed that these genes have close interactions. PCA found that the expression of these DEGs could well discriminate between ovarian cancer (blue) and normal (red) tissues (Fig. 1E). We extracted GO and KEGG pathway data for these genes based on the DAVID database. Regarding GO enrichment terms, these genes were enriched in cell adhesion molecule binding, actin binding, cadherin binding, actin filament binding, cell-substrate junction, cell-substrate adherens junction, focal adhesion, collagen-containing extracellular matrix, and antigen processing and presentation (Fig. 1F). Regarding KEGG enrichment terms, these genes were enriched in cell adhesion molecules, Staphylococcus aureus infection, haematopoietic cell lineage, viral myocarditis, and asthma (Fig. 1G). In summary, these results indicated that common DEGs highlighted the significant role of cell adhesion in the relationship between PCOS and OC.

### Evaluation of clinical outcomes in OC based on the 128 common DEGs

**Fig. 2:**
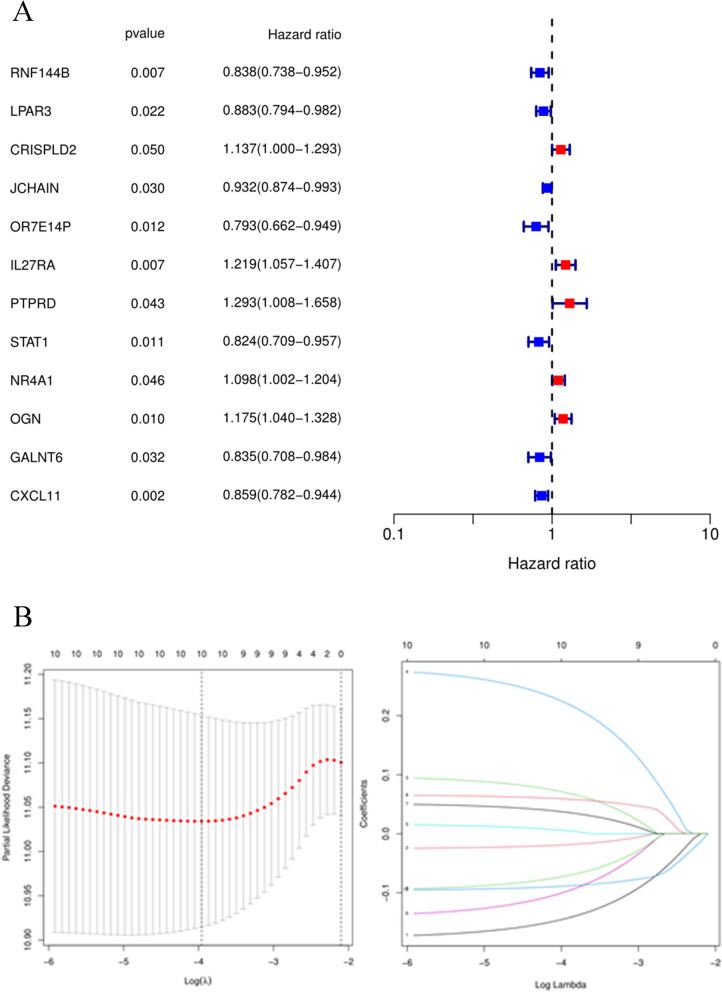
Key gene prognostic values. **A** Prognostic values of 12 genes based on forest plots. **B** Prognostic signature construction based on LASSO Cox analysis

The 128 common DEGs were used to analyse the prognosis in OC patients based on the univariate Cox method. A total of twelve key genes were closely associated with the prognosis of OC patients: RNF144B, LPAR3, CRISPLD2, JCHAIN, OR7E14P, IL27RA, PTPRD, STAT1, NR4A1, OGN, GALNT6 and CXCL11 (Fig. 2A). Then, we used these expression profiles to construct the prognostic model. The following risk score formula was developed: Risk score = RNF144B*(-0.1441) + LPAR3*(-0.0187) + CRISPLD2*0.0701 + IL27RA*0.2226 + PTPRD*0.0055 + STAT1*(-0.0988) + NR4A1*0.0369 + OGN*0.0590 + GALNT6*(-0.0718) + CXCL11*(-0.0886). Next, we divided these OC patients into high-risk and low-risk groups with the median risk score based on the risk score formula (Fig. 2B).

**Fig. 3:**
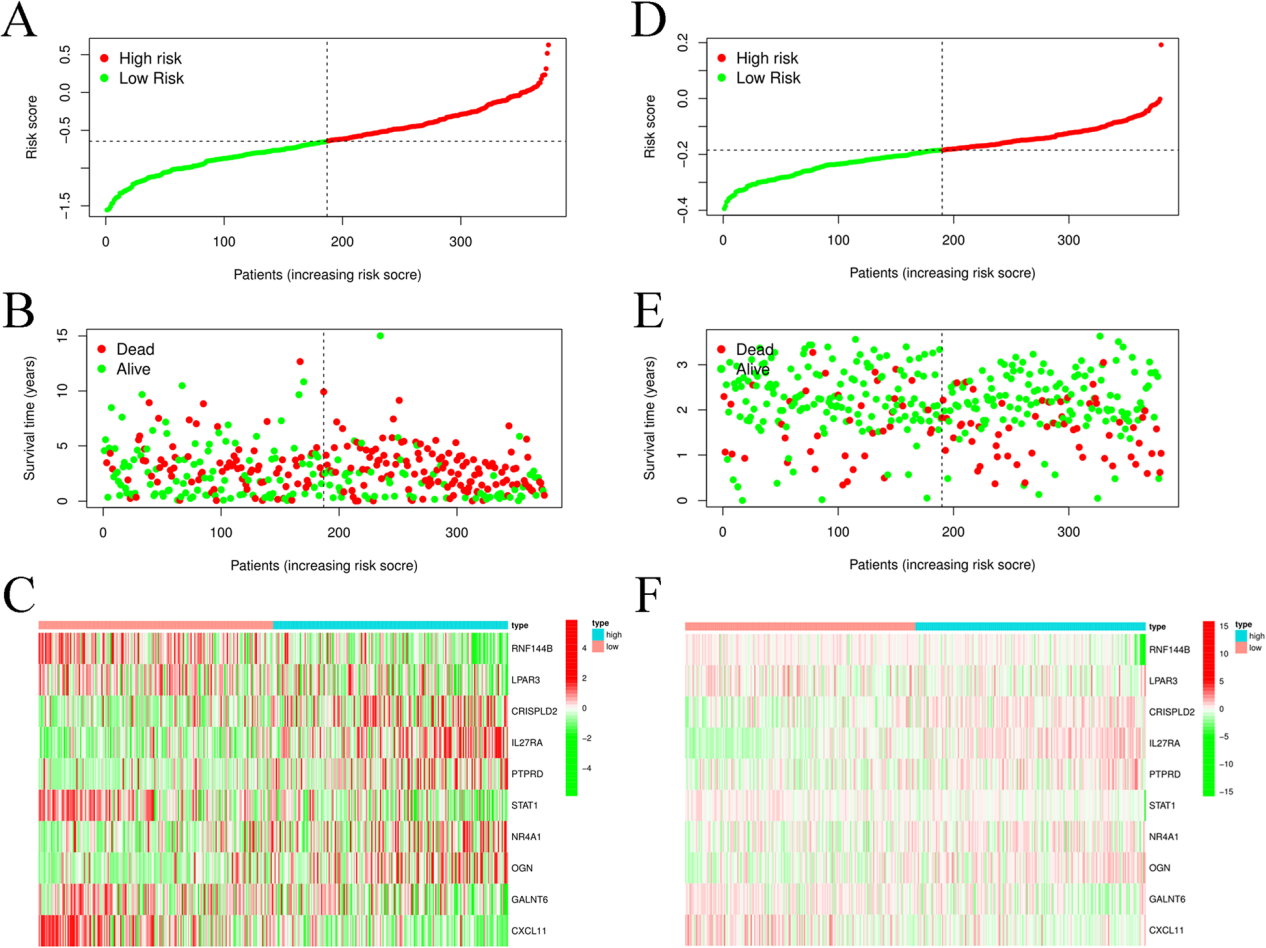
12 Prognostic index of OC patients. **A** The PI distribution of patients in the training dataset. **B** OC patient survival in the training dataset. **C** The expression profiles of 12 key genes in the training dataset. **D** The PI distribution of patients in the test dataset. **E**.OC patient survival in the test dataset. **F** The expression profiles of 12 key genes in the test dataset

The survival score and status of the two groups in the training cohort based on TCGA database OC datasets are shown in Fig. 3A&B. These twelve key gene expression profiles are shown in the heatmap (Fig. 3C). Moreover, we used the GSE140082 dataset as a test cohort to validate the risk score formula, and the survival score and status of the high-risk and low-risk groups are shown in Fig. 3D&E. These key gene expression files in the GSE140082 dataset were also visualized by a heatmap (Fig. 3F).

**Fig. 4:**
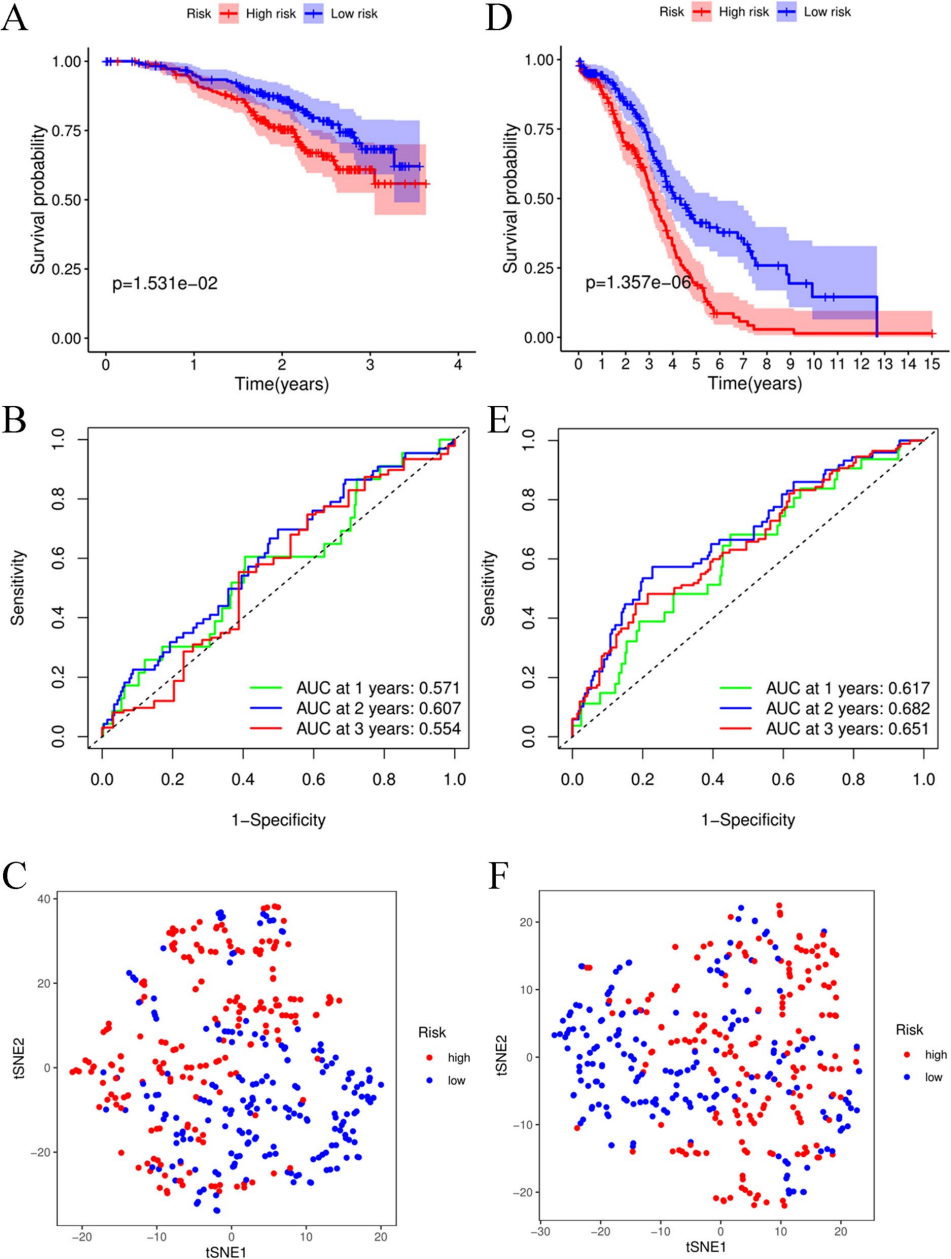
Prognostic analysis of the 12-gene signature model in the training cohort and test cohort. **A** Kaplan–Meier curves for the OS of patients in the high- and low-risk groups in the training cohort. **B** AUC time-dependent ROC curves for OS in the training cohort. **C** t-SNE analysis for OS in the training cohort. **D **Kaplan–Meier curves for the OS of patients in the high- and low-risk groups in the test cohort. **E** AUC time-dependent ROC curves for OS in the test cohort. **F **t-SNE analysis for OS in the test cohort

In the training cohort, the survival time and rate were significantly decreased as the risk score increased (Fig. 4A). The AUCs at 1, 2, and 3 years under the ROC curve were 0.571, 0.607, and 0.554, respectively, indicating that a moderate incubation period could be utilized as a prognostic marker of twelve key gene expression profiles in survival monitoring (Fig. 4B). However, t-SNE analysis showed that OC patients in different risk groups were not distributed in the two groups based on the TCGA database (Fig. 4C). To validate the efficiency of the prognostic model constructed from the TCGA-OC cohort, we used the median value of the training cohort to divide the OC patients from the GSE140082 cohort into high-risk and low-risk groups. Similar to the results of the training cohort, OC patients with high risk had a poor prognosis compared to other OC patients in the low-risk group (Fig. 4D). The AUC values at 1, 2, and 3 years were 0.617, 0.682, and 0.651, respectively, in the test cohort (Fig. 4E). In addition, t-SNE analysis results were similar to those noted for the training cohort (Fig. 4F), suggesting that the 12-gene signature could not be diagnostic markers for OC patients.

### The ectopic expression and prognostic significance of the 12-gene signature in OC patients

**Fig. 5:**
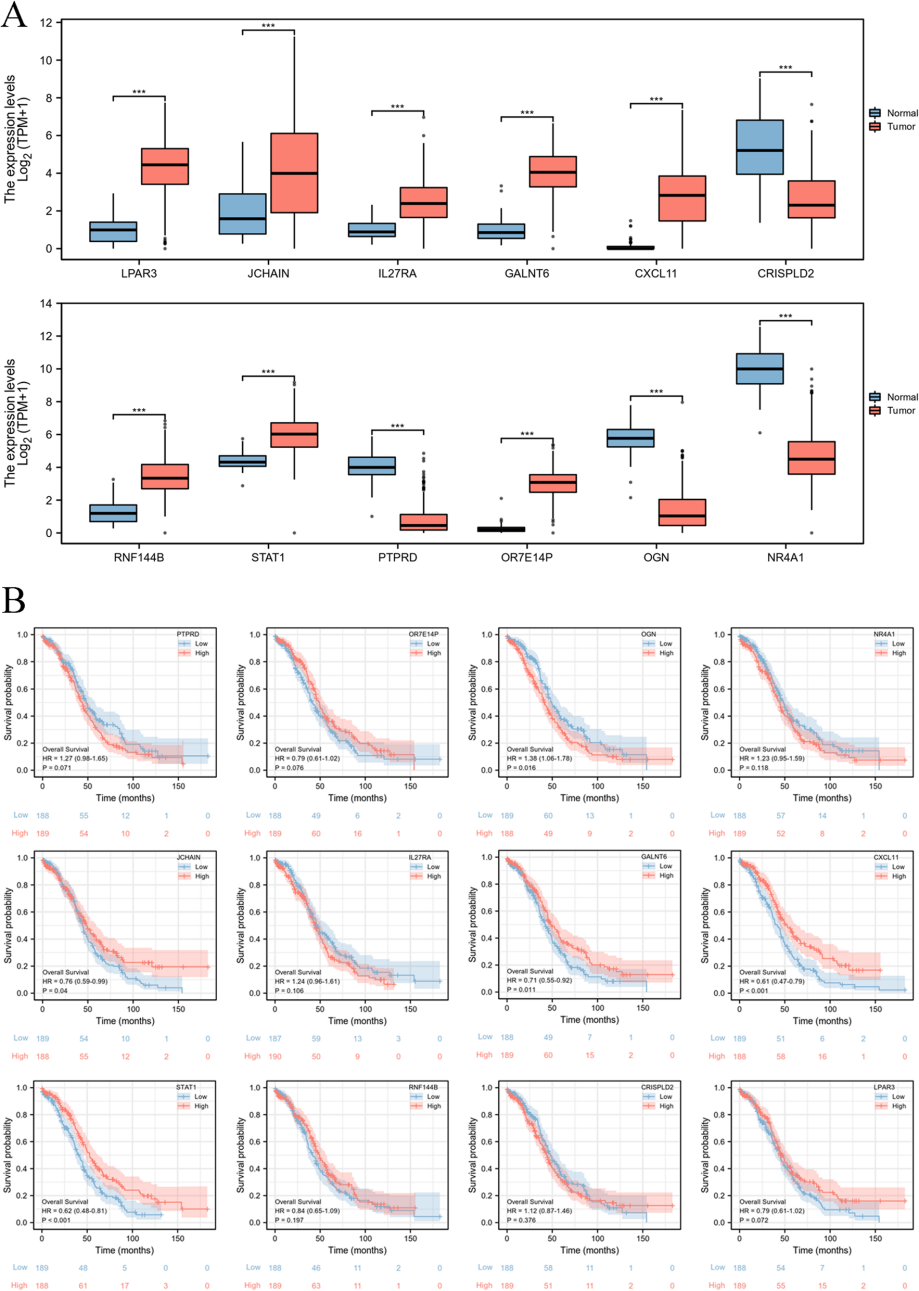
The expression and prognostic significance of the 12-gene signature. **A** The mRNA expression of the 12-gene signature based on TCGA database. **B** The prognostic significance of the 12-gene signature based on the TCGA database

Next, we used a boxplot to visualize the mRNA levels of the 12-gene signature in OC samples, indicating that LPAR3, JCHAIN, IL27RA, GALNT6, CXCL11, RNF144B, STAT1, and OR7E14P were significantly increased in OC patients, but CRISPLD2, PTPRD, OGN, and NR4A1 were obviously decreased in OC patients (Fig. 5A). We also confirmed the overall survival rate of the 12-gene signature in OC patients from TCGA database. The results suggested that OGN was significantly and negatively correlated with OC patient prognosis, but JCHAIN, GALNT6, CXCL11, and STAT1 were significantly and positively correlated with OC patient prognosis (Fig. 5B). These results suggested that JCHAIN, GALNT6, CXCL11, STAT1, and OGN might play a key role in OC patient progression.

### DNA alteration and immune infiltration of 5 key genes in OC progression

**Fig. 6:**
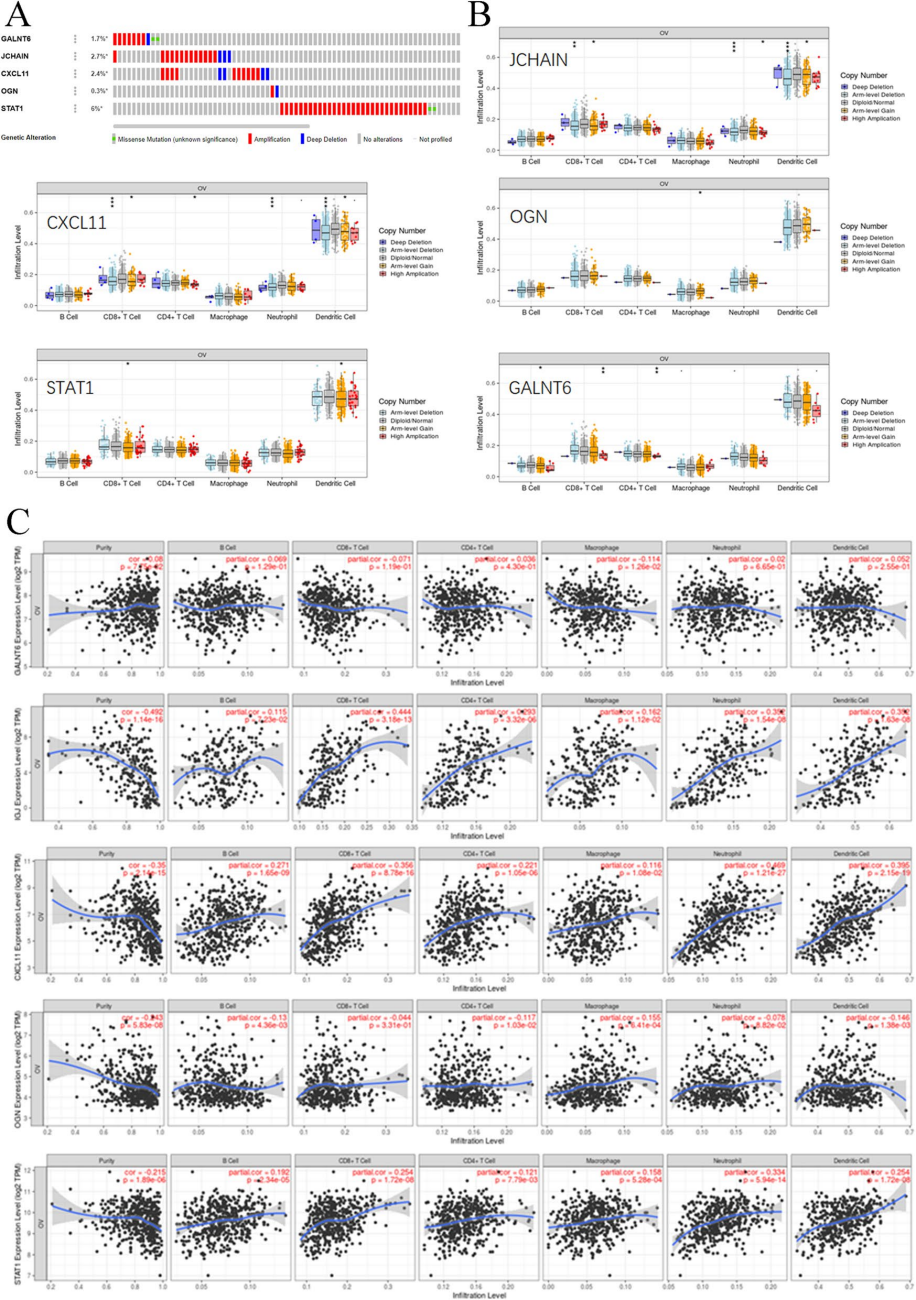
DNA alterations in and immune infiltration associated with 5 key genes. **A **DNA alterations in 5 key genes. **B** CNVs of the 5 genes. **C** Cancer purity and immune infiltration associated with 5 key genes

In Fig. 1F, we found that these common DEGs correlated with PCOS and OC and were significantly correlated with antigen processing and presentation, antigen processing and presentation of peptide antigens, antigen processing and presentation of peptide or polysaccharide antigens via MHC class II, and antigen processing and presentation of peptide antigens via MHC class II. These findings indicated that immune infiltration was closely associated with OC progression. We found that the 5 genes harboured genetic alterations, such as missense mutations, amplifications and deep deletions (Fig. 6A). Copy number variants (CNVs) of JCHAIN were significantly correlated with CD8 + T cells, neutrophils, and dendritic cells. CXCL11 CNVs were closely associated with CD8 + T cells, CD4 + T cells, neutrophils, and dendritic cells. The CNV level of OGN was markedly related to macrophages. STAT1 CNV levels were closely related to CD8 + T cells and dendritic cells. CNVs of GALNT6 were significantly associated with B cells, CD8 + T cells and CD4 + T cells (Fig. 6B). Furthermore, we found that GALNT6 mRNA expression was not obviously correlated with immune infiltration in any immune cell type. JCHAIN levels were closely associated with purity, CD8 + T cells, CD4 + T cells, neutrophils, and dendritic cells. CXCL11 expression was correlated with the infiltration of purity, B cells, CD8 + T cells, CD4 + T cells, neutrophils, and dendritic cells. The OGN level was significantly correlated with purity. STAT1 mRNA levels were closely related to purity, CD8 + T cells, neutrophils, and dendritic cells (Fig. 6C). Taken together, the expression and alteration of these 5 key genes were involved in the immune infiltration progression of OC.

### Drug sensitivity analysis of the hub genes

We further used drug sensitivity analysis to confirm these 5 key genes. The results showed that OGN was closely correlated with chemotherapy resistance based on the GSCALite database (Supplementary Fig. S1). Therefore, OGN could represent a potential target in the treatment of OC or PCOS patients.

### The characteristics of OGN in OC and PCOS

**Fig. 7:**
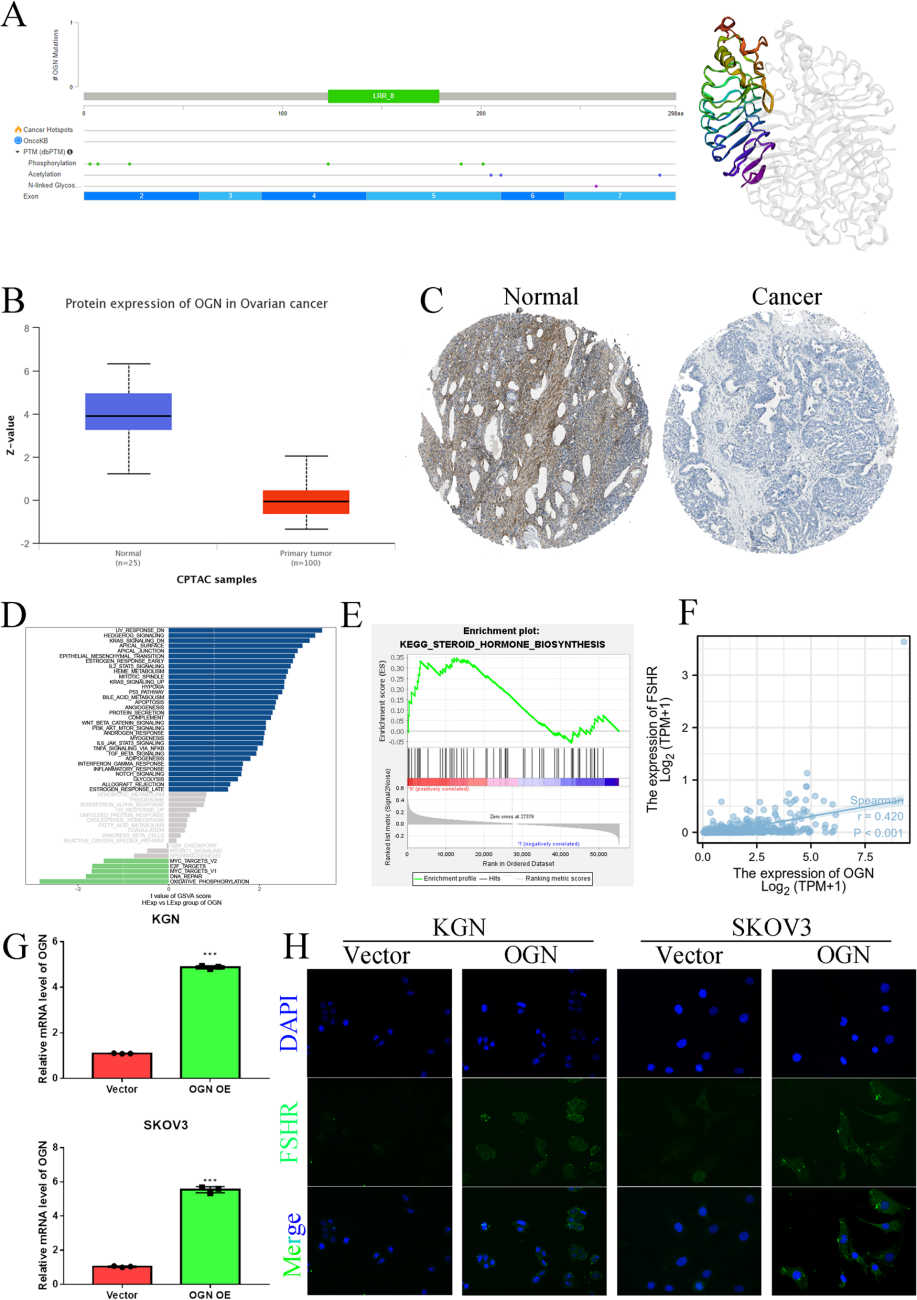
OGN structure, expression and function. **A** OGN structure. **B** OGN protein expression in OC based on the CPTAC database. **C** OGN protein expression in OC based on the HPA database. **D** GSVA analysis of OGN. **E** GSEA for OGN. **F** OGN and FSHR correlation analysis in TCGA-OC dataset. **G** The mRNA levels in vector- and OGN-overexpressing KGN and SKOV3 cells. **H** The effect of OGN on FSHR as assessed by immunofluorescence

To elucidate the expression, function and structure of OGN, we used the PDB database to confirm the OGN structure, as shown in Fig. 7A. OGN has an LRR_8 domain and multiple phosphorylation, acetylation and N-linked glycosylation sites. OGN protein expression was significantly decreased in OC tissue samples compared to normal ovary samples (Fig. 7B&C). We further utilized GSVA and GSEA to predict the potential function of OGN, as shown in Fig. 7D&E. OGN might be involved in steroid hormone biosynthesis and the steroid hormone response. Furthermore, we found that OGN levels were significantly and positively correlated with the level of FSHR in OC (Fig. 7F). We overexpressed OGN in KGN and SKOV3 cell lines (Fig. 7G) and confirmed the effect of OGN on FSHR expression by IF (Fig. 7H). In a previous study, an FSHR inhibitor restrained OC carcinogenesis by inhibiting the expression levels of FSHR, which has definite oncogenic potential and is a probable candidate for oncogenesis [25]. However, FSHR inhibitors can trigger a PCOS-like state [26]. These results suggested that the OGN/FSHR axis might play a dual role in the progression of OC or PCOS.

### OGN levels are correlated with regulators of ferroptosis and m6A methylation in OC

**Fig. 8:**
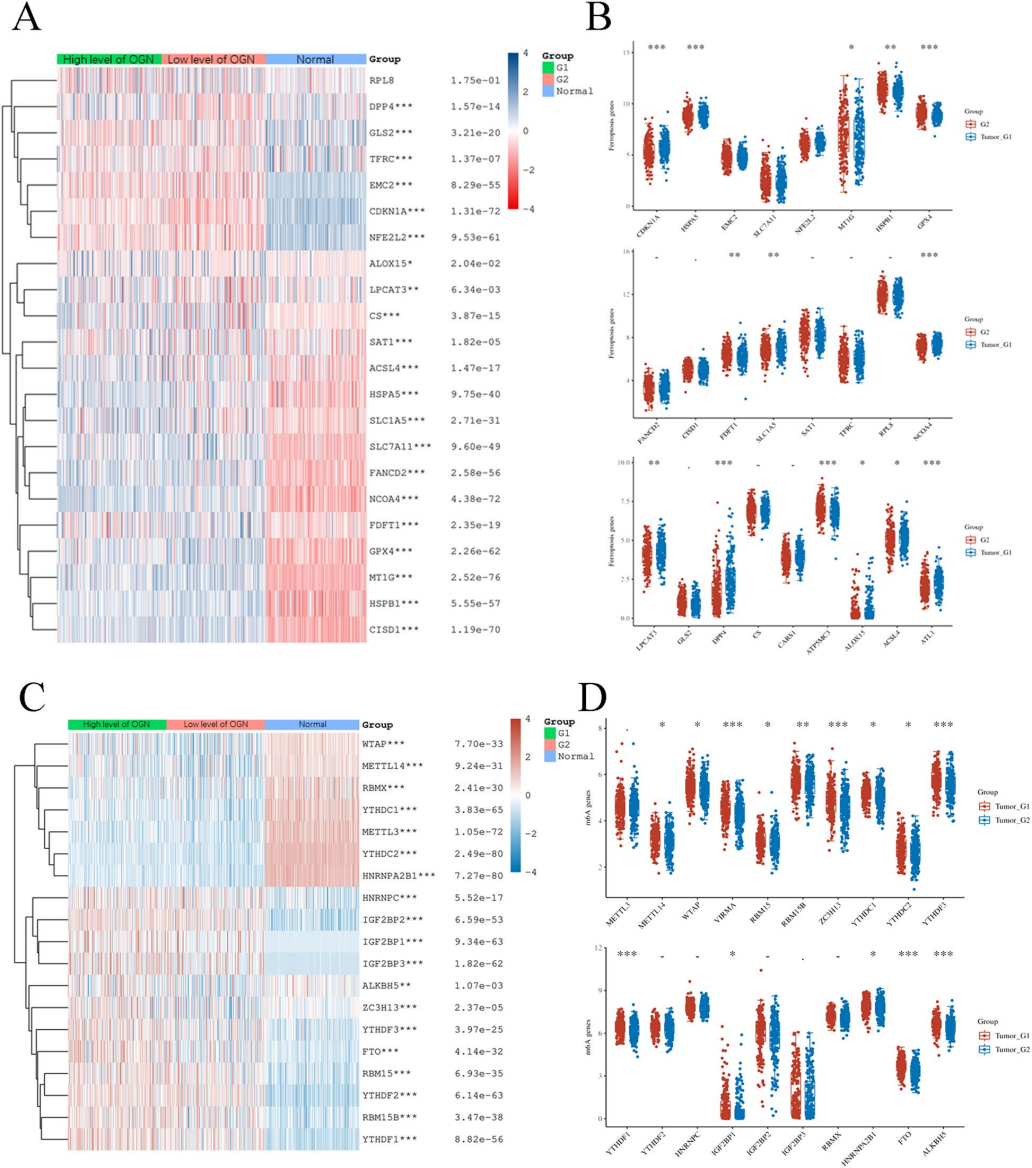
Association of OGN levels with ferroptosis- and m6A methylation-related genes in OC. **A** Ferroptosis gene expression in OC with high or low levels of OGN and normal ovaries. **B** Ferroptosis gene expression in OC with high or low levels of OGN. **C** m6A methylation gene expression in OC with high or low levels of OGN and normal ovaries. **D** m6A methylation gene expression in OC with high or low levels of OGN

Ferroptosis and m6A methylation are involved in the development and progression of OC. We first used the TCGA database to analyse whether OGN levels are correlated with ferroptosis. We performed a correlation analysis between OGN expression and 25 ferroptosis genes in OC and ovarian tissue samples based on TCGA and GTEx databases (Fig. 8A). The results showed that the expression of 25 ferroptosis genes was significantly different between OC and normal ovaries. Furthermore, we also confirmed the correlation of ferroptosis genes with OGN in OC samples. We found that OGN levels were positively correlated with MT1G, HSPB1, GPX4, FDFT1 and ATP5MC3 but negatively correlated with CDKN1A, HSPA5, SLC1A5, NCOA4, LPCAT3, DPP4, ALOX15, ACSL4, and ATL1 (Fig. 8B). Then, we extracted the expression profiles for the 20 m6A methylation genes between OC and normal ovary samples based on TCGA and GTEx databases (Fig. 8C). The results indicated that these m6A methylation regulators played a key role in OC progression. We further performed a correlation analysis between these m6A regulators and OGN expression. The results showed that METTL14, WTAP, VIRMA, RBM15, RBM15B, ZC3H13, YTHDC1, YTHDC2, YTHDF3, YTHDF1, IGF2BP2, HNRNPA2B1, FTO, and ALKBH5 were positively and significantly associated with OGN expression in OC patients (Fig. 8D). Taken together, these results suggest that OGN might have another important function in OC ferroptosis and m6A methylation modifications.

## Discussion

OC is a lethal malignancy in gynaecological diseases and is a complex disease, and multiple metabolic enzymes and pathways are involved in its pathophysiology [27]. PCOS is a benign gynaecological disease with multisystem metabolic disorders that are characterized mainly by HA, IR, LH and FSH ratio imbalance; infertility; endometrial disorder; obesity, and polycystic ovaries [1]. We found that these MRGs, such as ENPP1, FH, CYP2E1, HPGDS, ADCY9, NDUFA5, ADH1B and PYGB, were involved in the development and progression of OC, which could be used to construct a prognostic model to predict the postoperative risk for OC patients [17]. Zhou et al. also supported this view, and they found that PYGB could promote the development of OC by activating the Wnt/β-catenin signalling pathway [28]. Moreover, many studies have suggested that alternative AR activating signals, including both ligand-dependent and ligand-independent signals, are involved in OC progression [29, 30]. Therefore, the underlying pathophysiological association between PCOS and OC might be a key mechanism to target to formulate a clinical treatment strategy for OC or PCOS.

In this study, we found 128 shared DEGs between PCOS and OC compared to corresponding normal tissue samples. These 128 genes were significantly enriched in cell adhesion molecule binding via GO and KEGG analysis. Twelve genes, including RNF144B, LPAR3, CRISPLD2, JCHAIN, OR7E14P, IL27RA, PTPRD, STAT1, NR4A1, OGN, GALNT6, and CXCL11, were validated as key genes related to the prognosis of OC patients. A prognostic score based on the twelve genes obviously classified OC patients into high-risk and low-risk groups. Moreover, we found that the high-risk group had a poor prognosis compared to the low-risk group. Given the underlying molecular mechanisms of these MRGs, studies on the functions and mechanisms of RNF144B, JCHAIN, OR7E14P, IL27RA, PTPRD, and OGN have not been confirmed in OC progression. However, an additional six genes have been elucidated in OC development: LPAR3, CRISPLD2, STAT1, NR4A1, GALNT6, and CXCL11. LPAR3 expression is significantly increased in OC tissue samples compared to normal ovary tissue samples, and this gene might play a role in the carcinogenesis of ovarian cancer [31]. High CRISPLD2 expression was correlated with worse prognosis in OC patients [32]. High STAT1 levels were associated with improved prognosis in OC patients, and this information might be useful for the development of new immunomodulatory drugs for OC treatment [33]. Ectopic expression of NR4A1 protein was significantly correlated with poor prognosis in OC patients [34], potentially modulating platinum resistance in OC [35]. GALNT6 could modify O-glycans on EGFR to increase its activation, which was able to significantly enhance OC cell viability, migration, and invasion [36]. Many studies have indicated that CXCL11 promotes OC progression by mediating angiogenesis [37], lymph node metastasis [38], and immune infiltration [39, 40]. Moreover, RNF144B, JCHAIN, OR7E14P, IL27RA, PTPRD, OGN, CRISPLD2, and GALNT6 have not been confirmed in PCOS progression. STAT1 interacts with the CD44-OPN adhesion complex, ERα, and NF-κB to formulate significant crosstalk, resulting in the modulation of endometrial receptivity [41]. Insulin enhances STAT1 and STAT3 expression to repress the levels of miR-27a-3p, which could decrease granule cell proliferation and apoptosis escape [42]. High androgen levels could mediate a series of important genes, including TFAP2A, ETS1, ELK1, ERG, FLI1 and SPI1, to increase NR4A1 levels in PCOS [43]. CXCL11 expression was significantly and obviously correlated with prolactin and 17-OH-progesterone levels in PCOS [44]. Based on previous studies and our study, we were able to obtain only limited information about the 12 key genes involved in OC patient survival and pathophysiological changes in PCOS patients.

To seek efficient therapeutic agents for the treatment of OC and PCOS, we further screened these 12 key genes based on expression, survival significance, immune infiltration, and drug sensitivity. Osteoglycin (OGN), a small proteoglycan with tandem leucine-rich repeats (LRR), is overexpressed in blood vessels and bone, modulating bone formation mediated by transforming growth factor beta [45]. OGN is involved in the progression of extracellular matrix and remodelling and tissue development [45, 46]. Katja Hummitzsch et al. found that OGN mRNA expression was significantly upregulated in the internal ovary theca compared to the stroma [47]. Hao and his colleagues further found that OGN protein expression was markedly associated with signalling pathways related to follicular development, especially the oestrogen, insulin, and PI3K-Akt signalling pathways [48]. We found that OGN mRNA and protein expression were significantly decreased in OC/PCOS compared to normal ovary tissues or granule cells from women without PCOS. Taken together, these results indicated that OGN might represent a significant factor in OC and PCOS progression. Furthermore, our GSVA and GSEA showed that highly expressed OGN could also enhance steroid hormone biosynthesis, and correlation analysis indicated that OGN was significantly associated with FSHR levels. Many studies have indicated that FSHR is a significant oncogene in OC development that can mediate cell proliferation [49, 50], metastasis [51], and apoptosis escape [52]. Moreover, FSHR levels are significantly decreased in PCOS granulosa cells compared to normal granulosa cells [53]. FSHR is critical for FSH-mediated follicle growth and development, and a decrease in the FSH/FSHR pathway might induce follicular growth arrest, promoting the progression of PCOS [54, 55]. Therefore, OGN might upregulate FSHR to sensitize the steroid hormone response, which could accelerate OC formation and progression but reverse PCOS progression. Low OGN expression is an important feature from PCOS to OC, indicating that PCOS patients with low levels of OGN may have a greater OC risk. Aberrant OGN activation is associated with high tumour invasiveness and poor prognosis in OC patients [56]. Moreover, the function of OGN in OC progression is closely related to m6A modification and ferroptosis. Ferroptosis is a novel recognized type of cell death induced by iron accumulation, lipid peroxidation and glutathione deprivation, indicating a connection with a variety of disorders and suggesting great potential in OC therapy [57]. The ectopic expression of m6A methylation regulators is involved in OC progression. Specifically, YTHDF1, YTHDF2, IGF2BP1, METTL3, ALKBH5, WTAP, and FZD10 were upregulated, whereas FTO was downregulated [58]. Taken together, these results suggest that OGN might regulate m6A methylation or ferroptosis to promote OC concurrence, development, and progression. In summary, we infer that OGN might represent a new risk marker for PCOS to OC; however, this assumption needs to be verified in further studies.

## Conclusions

In conclusion, this study provided evidence about the association between PCOS and OC. Through the functional analysis of identified DEGs, we found that cell adhesion was significantly enriched in the PCOS and OC datasets. We also confirmed 12 key genes to construct a prognostic model that classified OC patients into high-risk and low-risk groups for prognostic prediction. Moreover, we found that OGN might represent a key biomarker, indicating a greater OC risk in PCOS patients. Nevertheless, further experimental verification is required.

## Supplementary Information

**Additional file 1.**

## Data Availability

The data used to support the findings of this study are available from the corresponding author upon request.

## Abbreviations

PCOS: Polycystic ovary syndrome
HA: Hyperandrogenemia
IR: Insulin resistance
LH: Luteinizing hormone
FSH: Follicle-stimulating hormone
OC: Ovarian cancer
TCGA: The Cancer Genome Atlas Gene
GEO: Expression Omnibus
EC: Endometrial cancer
BC: Breast cancer
MRGs: Metabolism related genes
PPI: Protein–protein interactions
OGN: Osteoglycin
