# Supplementary figures and images for: Identification of key genes associated with polycystic ovary syndrome (PCOS) and ovarian cancer using an integrated bioinformatics analysis
Source: J Ovarian Res. 2022 Feb 28;15:30. doi: 10.1186/s13048-022-00962-w (PMC8886837; doi:10.1186/s13048-022-00962-w)

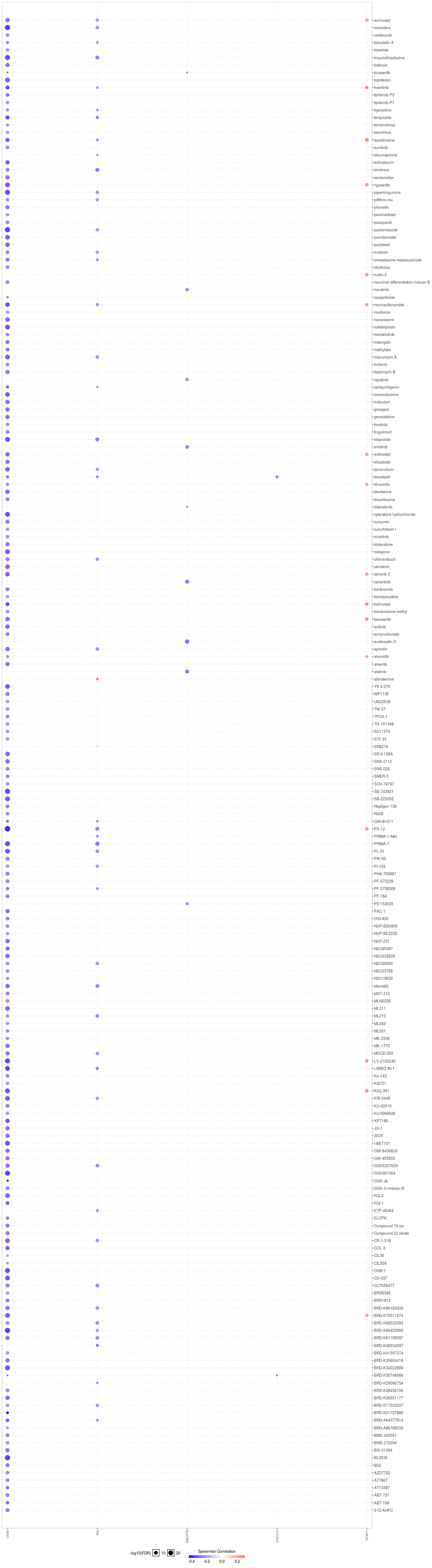

Supplement: Supplementary file 1 — Additional file 1. [file 13048_2022_962_MOESM1_ESM.png]
